# Integrating fine needle aspiration and single-cell RNA sequencing for studying metabolic dysfunction-associated steatotic liver disease

**DOI:** 10.3389/fmed.2026.1817738

**Published:** 2026-07-20

**Authors:** Matthew P. Salomon, Lucy Golden-Mason, Ivetta Vorobyova, Gary C. Kanel, Yufen Wang, Daphne Wong, Ana C. Maretti-Mira

**Affiliations:** 1 Department of Cancer Biology, Keck School of Medicine, University of Southern California, Los Angeles, CA, United States; 2 Division of Gastrointestinal and Liver Diseases, Department of Medicine, Keck School of Medicine, University of Southern California, Los Angeles, CA, United States; 3 Molecular Imaging Center, Department of Radiology, Keck School of Medicine, University of Southern California, Los Angeles, CA, United States; 4 Department of Pathology, Keck School of Medicine, University of Southern California, Los Angeles, CA, United States

**Keywords:** immunology, MASH, MASLD, molecular biology, new approach methodology

## Abstract

**Introduction:**

Metabolic dysfunction-associated steatotic liver disease (MASLD) is currently the leading cause of chronic liver disease and hepatocellular carcinoma. The immune response plays a central role in disease onset and progression and is the focus of many experimental studies. However, traditional models typically rely on terminal sampling procedures that require large tissue quantities, a substantial number of animals per experimental condition, and cross-sectional study designs.

**Methods:**

Here, we propose integrating two powerful techniques to longitudinally study a MASLD animal model: image-guided fine-needle aspiration (FNA) and single-cell RNA sequencing (scRNA-seq).

**Results:**

This framework enables safe, high-precision longitudinal sampling of a limited number of animals, allowing simultaneous profiling of innate and adaptive immune cells during metabolic dysfunction-associated steatohepatitis (MASH) progression induced by a high-fat, high-cholesterol, and high-fructose diet. We further identified dynamic shifts in the hepatic immune landscape following dietary intervention.

**Conclusion:**

Our findings support the feasibility of the FNA–scRNA-seq framework as a novel tool for longitudinal immune profiling in preclinical MASLD and MASH studies and highlight its potential to reduce animal use in chronic liver disease research.

## 1 Introduction

Metabolic dysfunction-associated steatotic liver disease (MASLD) is the most prevalent chronic liver disease worldwide, affecting approximately 34% of adults in the United States (1, 2). MASLD encompasses a disease spectrum ranging from simple steatosis to metabolic dysfunction-associated steatohepatitis (MASH) (3). Sterile inflammation plays a pivotal role in MASH pathogenesis, driven by lipid-induced hepatocyte stress and death and by complex interactions between innate and adaptive immune cells, which together promote tissue injury (4). In severe cases, MASH can further progress to cirrhosis and ultimately hepatocellular carcinoma (HCC) (5). Current experimental approaches for studying MASLD rely heavily on animal models, particularly rodents (6). Recently, several agencies, including the National Institutes of Health (NIH), have actively supported the development and implementation of new approach methodologies (NAMs) that focus on human-relevant models, aiming to reduce and replace animal use whenever possible in biomedical research (7). Although *in vitro* complex models, such as human 3D cell cultures and human liver-like organ technologies, show promising results for advancing MASLD/MASH studies, animal models remain essential for complementary insights into systemic interactions that are essential for disease development.

Fine-needle aspiration (FNA) under image guidance is a well-known diagnostic procedure of choice for patients with focal hepatic lesions and masses (8). It is a simpler, less invasive, and painless tissue-collection technique than biopsy (9), yet remains underexplored as a tool for animal studies. The FNA procedure is an effective alternative to biopsy for following fungal inoculation in mice (10). Additionally, FNA has been used in a few experimental cancer studies. It has been paired with flow cytometry to identify and quantify liver-solid-tumor-infiltrating cell subsets in mice (11) and to collect cells from subcutaneous tumors for scRNA-seq (12). However, studies in non-tumor tissues, particularly the liver, are lacking and could help assess the feasibility of this approach for MASLD modeling.

To address this knowledge gap, we propose an innovative approach in which we longitudinally sampled diet-induced MASLD mice using image-guided FNA targeting major liver lobes. The harvested tissues were digested to preserve hepatic immune cells, crucial drivers of the disease progression, which were then profiled using single-cell RNA sequencing (scRNA-seq). Our study focused on the role of dietary cholesterol in the context of a high-fat, high-fructose diet in the development of MASH, and on the potential impact of dietary intervention on reversing inflammation. This approach captured the hepatic immunological landscape during MASH progression, extracting disease-relevant information while using fewer animals. Our study supports the feasibility of the FNA-scRNAseq framework as a new tool for longitudinal immune profiling in MASLD and for reducing animal testing in chronic liver disease studies.

## 2 Materials and methods

### 2.1 Murine model of diet-induced MASH

This disease model has been previously described by our group (13). Briefly, seven-week-old male wild-type C57BL/6 J mice (Jackson Laboratory) were fed the well-established Fructose, Palmitate, Cholesterol, and Trans-Fat (FPC) diet (Envigo) to induce MASH. Our study consisted of two phases. The first phase, the Progression phase, lasted 24 weeks. During this phase, five mice received an FPC diet with low cholesterol (0.05%), and five received an FPC diet with high cholesterol (1.25%). At the end of this phase, three mice underwent the fine-needle aspiration (FNA) procedure and were carried to the next study phase, while the two remaining mice were euthanized, and their livers were used for histological assessment. Samples collected in this phase from the low-cholesterol group were labeled as P-LC24, and samples from the high-cholesterol group were referred to as P-HC24. In the following phase, the Intervention phase, all mice were fed an FPC diet containing low cholesterol (0.05%) for an additional 8 weeks, totaling 32 weeks of study. At the end of this phase, all mice underwent the FNA procedure and were then euthanized for histology. Samples collected from mice continuously fed a low-cholesterol diet were labeled as I-LC32, and samples from the other group were labeled I-HC32. This study was reviewed and approved by the University of Southern California Institutional Animal Care and Use Committee.

### 2.2 Fine-needle aspiration (FNA) procedure

Mice were maintained under anesthesia throughout the procedure by continuous inhalation of isoflurane (1–4% in oxygen). Hair over the imaging site was removed with Nair, and the area was cleaned with alcohol before the FNA procedure. Eye lube was applied to the eyes, and the ultrasound stage was heated and equipped with ECG and temperature monitoring. The ultrasound gel was applied to the prepared area, and the transducer was positioned over the liver to visualize the collection site. FNA was performed with a 0.60 × 25 needle coupled to a 10 mL syringe, which was attached to a hand-grip syringe holder. The procedure consisted of introducing the needle into the tissue and applying suction while moving the needle back and forth repeatedly. Enough material was considered obtained when the hub of the needle was filled with aspirate, the pressure on the syringe was released, and the needle was removed from the lesion. We harvested tissue from the 4 largest lobes of the liver (Right Lobe, Left Lobe, and 2 Middle Lobes). FNAs were placed in RPMI + 10% FBS and kept on ice until processing. A slight pressure for 10–15 s (cloth forming time) was applied to the collection site after each FNA as a precaution against hemorrhage development. After the procedure, animals were placed in a clean cage for recovery and monitored every 10 min for 2 h.

### 2.3 Liver histology

Animals used for liver histology were euthanized by CO₂ asphyxiation (30% flow rate until respiration ceased, followed by 70% for 2 min), and death was confirmed by cervical dislocation. Liver fragments from each lobe were harvested and fixed in 10% neutral buffered formalin overnight, followed by dehydration in 70% ethanol at 4 °C. After paraffin embedding, fragments were sectioned (5 μm thickness), deparaffinized, hydrated, and stained with H&E and Sirius Red at the USC Research Center for Liver Diseases (RCLD) Liver Histology Core. Liver biopsies from 4 lobules were evaluated and graded histologically as follows: Steatosis grades: “0” = none, “<1” = less than 5% of hepatocytes, “1” = 5–25%, “2” = 26–50%, “3” = 51–75%, “4” = > 75%; Lobular inflammation grades (20x field): “0” = none, “1” = < 2 per field, “2” = 2–4 per field, “3” = > 4 per field; Fibrosis grades: “0” = None, “1” = Perisinusoidal or periportal, “2” = Perisinusoidal and portal/periportal, “3” = Bridging fibrosis, “4” = Cirrhosis. The scoring was performed by a pathologist (G.K.) in a blinded fashion, without knowledge of the treatment.

All statistical analyses, and graphs were generated using GraphPad Prism version 10 for macOS (GraphPad Software). We used the non-parametric Kruskal-Wallis test to evaluate hepatic steatosis, inflammation and fibrosis scores across groups.

### 2.4 FNA biopsy cell dissociation

FNA biopsies from all animals within the same subgroups (a total of 12) were pooled into two 15 mL conical tubes and centrifuged at 350 rcf for 5 min at 4 °C. We gently resuspended the biopsies in 10 mL of liver digestion buffer per tube, preheated to 37 °C. Liver digestion buffer was composed of 0.05% Collagenase type IV (Cat#17104–019, Gibco) and 0.02% DNase (Cat#DN25, Sigma-Aldrich) in RPMI 1640 (Cat#22440, Invitrogen). Biopsies were dissociated into a single-cell suspension for 15 min at 37 °C on a rocker. The two 15 mL tubes were combined into a 50 mL conical tube, and the tube volume was filled with RPMI containing 10% FBS. Cells were centrifuged at 350 rcf for 5 min at 4 °C. Cell pellet was resuspended in 40 mL of 1X BD Pharm Lyse™ Lysing Buffer (Cat#555899, BD Biosciences) to remove red cells and incubated for 10 min at room temperature. Cell suspension was then filtered through a 40 μm cell strainer to remove debris and centrifuged at 350 rcf for 5 min at 4 °C. The cell pellet was resuspended in 30 mL of PBS and centrifuged at 350 rcf for 5 min at 4 °C. Cell pellet was resuspended in the Dead Cell Removal Kit microbead solution (Cat#130–090-101, Miltenyi Biotech), and dead cell removal was performed according to the manufacturer’s instructions. After viable cells were recovered, they were centrifuged at 400 rcf for 10 min at 4 °C, and the pellet was resuspended in 50 μL of PBS + 0.04% BSA. Cell concentration and viability were assessed using trypan blue exclusion. All samples showed viability above 95%.

### 2.5 Single-cell RNA library preparation and sequencing

An average of 16,500 cells per condition were partitioned using the Chromium Next GEM Single Cell 3′ v3.1 Kit (Cat#1000269, PN-1000130 and PN-1000129, 10XGenomics), targeting 10,000 cells. One scRNA-seq library per experimental condition was constructed following the manufacturer’s instructions (Cat#1000269, PN-1000196, 10X Genomics). Libraries were deep sequenced at the USC Molecular Genomics Core using the Illumina NovaSeq6000 platform, and an average of 40,000 reads was obtained per cell.

### 2.6 Bioinformatics analyses

Cell-by-gene count matrices were generated by processing FASTQ files with the Cell Ranger count pipeline (versions 9.0.1 and 10X Genomics) using the GRCm39 mouse reference genome (GRCm39-2024-A, 10X Genomics) for each sample. Count matrices were corrected for ambient RNA contamination using SoupX with default parameters (14). Quality control was performed using Seurat (version 5.3) (15) in a two-step process. First, genes detected in fewer than 3 cells were removed. Second, low-quality cells were filtered by removing cells with fewer than 500 UMIs, fewer than 300 detected genes, and greater than 10% mitochondrial reads (Supplementary Figure 1).

Samples were integrated into a unified data set using sctransform normalization (16, 17) and Harmony (version 1.2.3) integration (18) implemented in Seurat. Harmony integration was performed on the principal component embedding with each scRNA-seq library treated as a separate batch to correct the library-level technical variation across the four samples. No biological covariates were included in the correction. Integration performance was assessed by comparing UMAP embeddings before and after Harmony, colored by condition (Supplementary Figure 2). Differences in mitochondrial gene content among cells were corrected during normalization by regressing out the percentage of mitochondrial reads using the vars.to.regress parameter in the SCTransform function. Clustering was performed using the first 30 PCs as input dimensions for the Seurat FindNeighbors function. Optimal clustering resolution was determined by evaluating a range of values from 0 to 1 (incremented by 0.1) using the R package clustree (version 0.5.1) (19) and a final resolution of 0.4 was selected for downstream analysis. Cell types were annotated manually by examining the expression of canonical marker genes across each cluster (Supplementary Figure 3). Clusters were embedded in two-dimensional space using UMAP and visualized in Seurat (Supplementary Figure 4).

Differential expression analysis was performed at two levels. First, in the global comparison, all cells from one condition were compared against all cells from the contrasting condition. Second, in the cell-type-stratified comparison, cells of a given annotated cell type from one condition were compared against cells of that type from the contrasting condition. Genes were pre-filtered for testing using Seurat’s default thresholds (logfc.threshold = 0.25 and min.pct = 0.1). *p*-values were adjusted for multiple comparisons using Bonferroni correction across all tested genes, and genes with an adjusted *p*-value < 0.1 were considered differentially expressed.

DEGs included for biological interpretation had FDR < 0.1 and fold change > 1.2. Ingenuity Pathway Analysis (IPA) software (V153384343, Qiagen) (20) was used to determine the canonical pathways and biological processes altered at both global and cell-type-specific levels. The significance thresholds for pathways and biological processes were *p*-value <0.05 and Z-score <−2 or >2. Values used for data visualization were-log10(p-value) and Z-score. For visualization purposes, significant IPA pathways were grouped according to their primary biological functions. DEGs that shifting their direction of expression following dietary intervention were analyzed using the Gene Ontology website (21) (GO Ontology database DOI: 10.5281/zenodo.16423886 Released 2025-07-22). We used “*Mus musculus* (all genes in database)” as the reference list and “GO biological process complete” as the annotation data set. We selected Fisher’s exact test for statistical analysis and applied false discovery rate (FDR) correction for multiple testing. The threshold for significant biological processes was FDR < 0.05. Values used for data visualization were FDR and fold enrichment.

## 3 Results

### 3.1 Feasibility of longitudinal liver sampling using image-guided FNA and scRNA-seq

**Figure 1:**
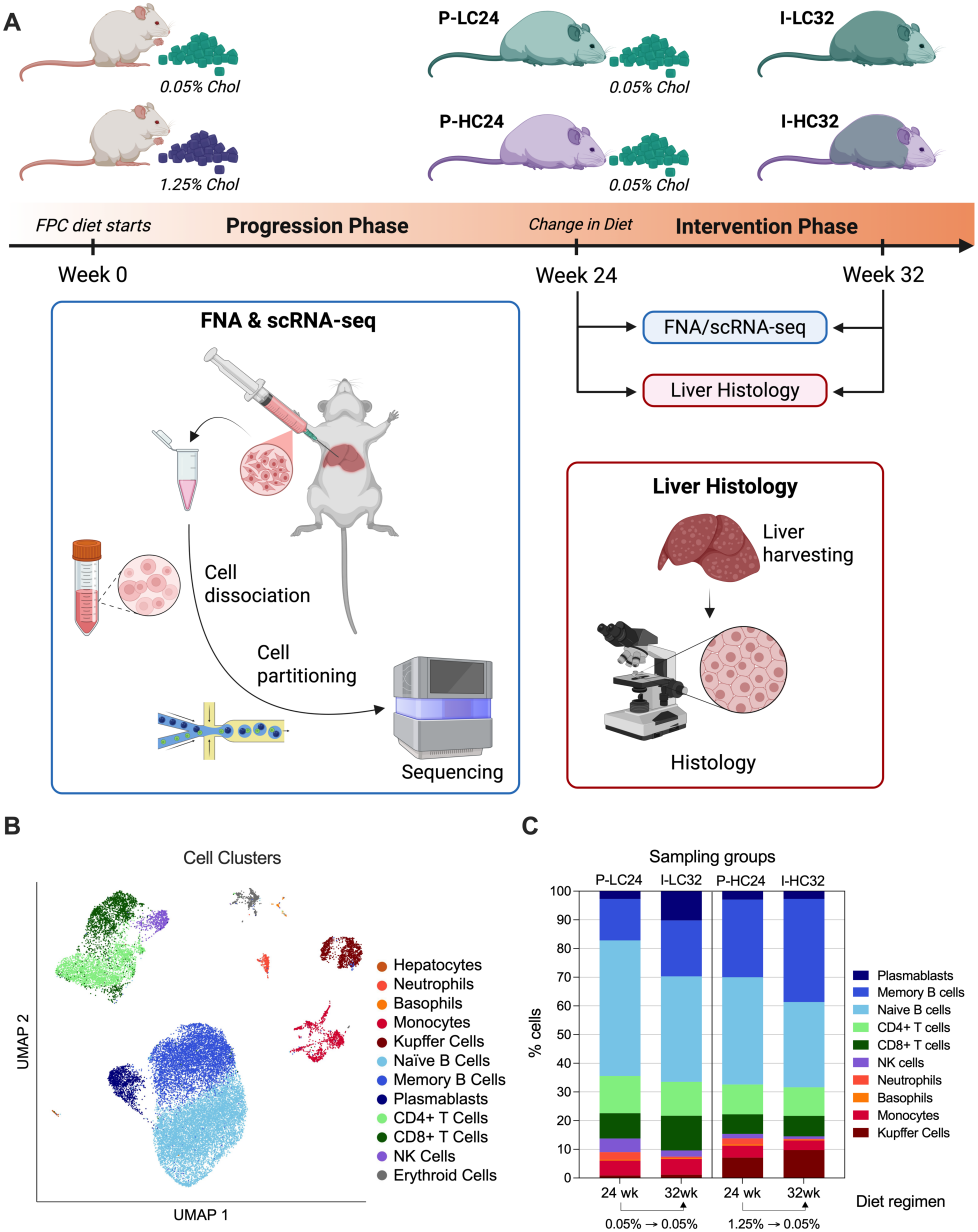
Use of image-guided FNA and scRNA-seq for longitudinal sampling in a MASLD animal model. **(A)** Experimental design. Mice were fed an FPC diet containing either low (0.05%) or high (1.25%) cholesterol for 24 weeks (progression phase). Three animals were randomly selected to undergo the FNA procedure and were subsequently fed an FPC diet containing low cholesterol for an additional 8 weeks (intervention phase), while the other two mice were euthanized for histological assessment. At the end of 32 weeks, all remaining mice underwent the FNA procedure and were then euthanized for histological analysis. Biopsies collected during the FNA procedure were dissociated into single-cell suspensions and analyzed by scRNA-seq. Created with www.biorender.com. **(B)** UMAP showing cell populations identified in FNA biopsies by scRNA-seq. **(C)** Proportions of cell populations observed across sampling groups.

To assess the feasibility of high-precision sampling using image-guided fine-needle aspiration (FNA) coupled with single-cell transcriptomics (scRNA-seq) in MASLD modeling, we applied this framework to evaluate the impact of dietary intervention on MASH progression (Figure 1A). This approach enabled longitudinal sampling of the same animal cohorts across disease stages while reducing the need for large tissue inputs and euthanasia typically required for traditional assays.

Two groups of mice (n = 5 each) were fed the Fructose, Palmitate, Cholesterol, and Trans-Fat (FPC) diet with varying cholesterol content for a total of 32 weeks (Figure 1A). During the first 24 weeks (Progression phase), one group received a low dietary cholesterol level (0.05%), and the other received a high dietary cholesterol level (1.25%). At week 24, three mice from each group underwent the FNA sampling and continued into the second phase of the study, while two mice from each group were euthanized for liver histological assessment. Samples harvested at week 24 from these groups are referred to as P-LC24 and P-HC24, respectively. All mice undergoing FNA recovered fully without complications. The remaining mice (three per group) were then transitioned to an FPC diet with 0.05% cholesterol for an additional 8 weeks (Intervention phase). At the end of this phase (week 32), all animals underwent a second FNA procedure and were subsequently euthanized for histological evaluation. Samples collected at this point were labeled I-LC32 (mice continuously fed low cholesterol) and I-HC32 (mice transitioned from high to low cholesterol during intervention). MASLD progression was confirmed using H&E and Sirius Red staining. No significant differences were detected in steatosis, inflammation, and fibrosis among the groups (Supplementary Table 1).

To obtain a better representation of the whole liver, we collected one biopsy per major liver lobe during each FNA procedure, totaling 4 biopsies per animal. Biopsies were pooled at the subgroup level for downstream cell dissociation. We obtained a robust number of viable cells for scRNA-seq (Supplementary Table 2). After quality control filtering and doublet removal (Supplementary Figure 1), we profiled 5,132 cells in P-LC24, 6,897 cells in P-HC24, 7,405 cells in I-LC32, and 7,343 cells in I-HC32. We identified Kupffer cells (KCs), monocytes, granulocytes, natural killer (NK) cells, and lymphocytes (Figure 1B). Since the dissociation procedure preferentially preserves hepatic non-parenchymal/immune cell population, only a very small number of hepatocytes and other cell types were recovered; these were excluded from cell population analysis. Gene markers used for cell type annotation are provided in Supplementary Figure 3. We observed shifts in immune cell composition driven by dietary cholesterol and exposure duration (Figure 1C). Notably, mice fed high cholesterol for 24 weeks exhibited an increased proportion of KCs and memory B cells compared to their low cholesterol counterparts. Dietary intervention did not reduce KC abundance.

**Figure 2:**
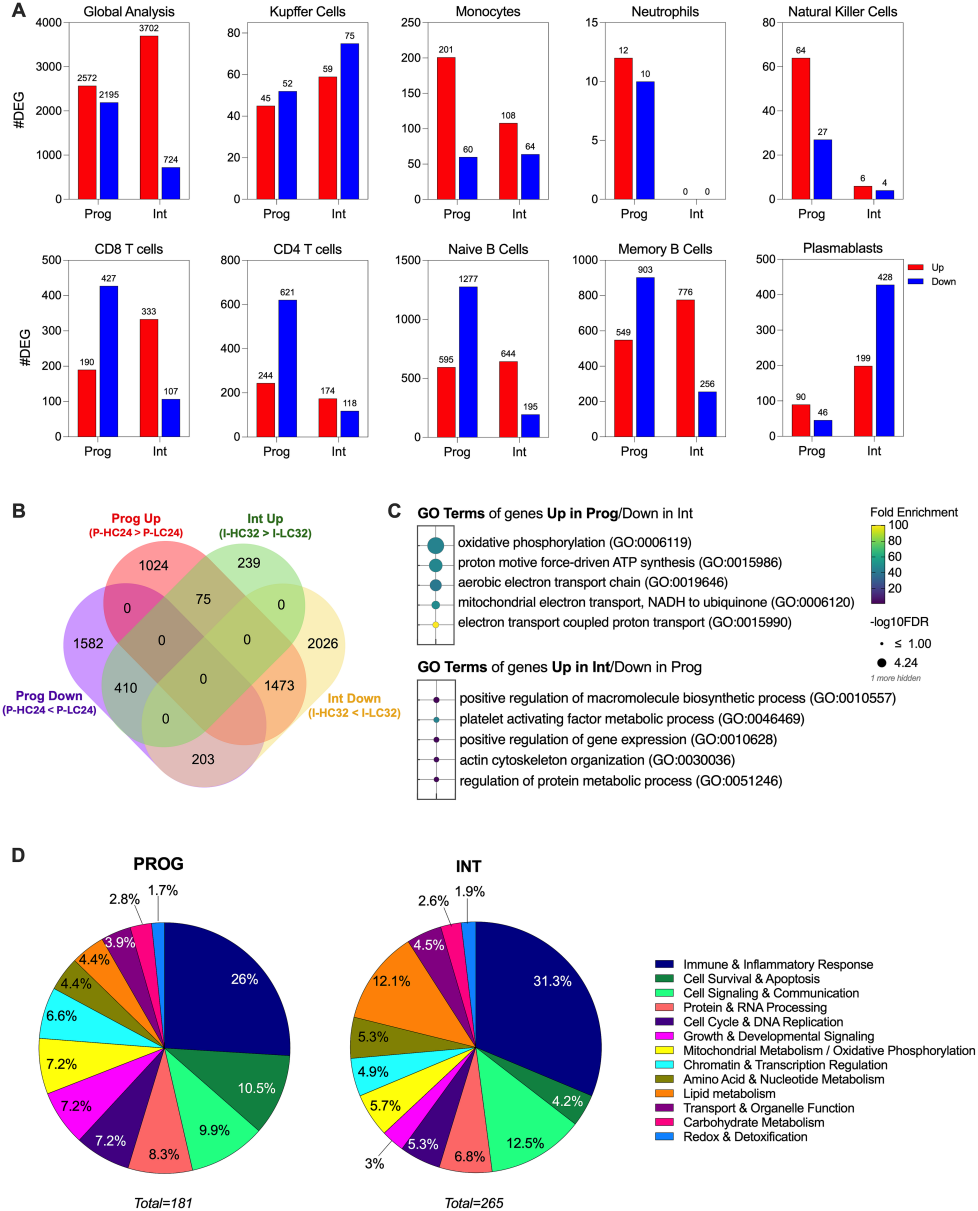
Dietary cholesterol impact on hepatic immune cell transcriptomes. **(A)** Differentially expressed genes (DEGs) were identified using both global analysis and cell type–specific analyses with the pairwise comparisons: P-HC24 versus P-LC24 (Progression, Prog), and I-HC32 versus I-LC32 (Intervention, Int). **(B)** The Venn diagram illustrates the overlap of upregulated and downregulated genes at the global level in the Progression and Intervention groups. **(C)** Bubble plots visualize the Gene Ontology (GO) biological processes enriched by genes that shift their direction of expression regulation after cholesterol reduction. The top panel shows enrichment based on the 75 genes upregulated during progression phase, whereas the bottom panel shows enrichment based on 203 genes upregulated after dietary intervention. **(D)** Pie charts depict the distribution of significant biological processes identified by Ingenuity Pathway Analysis (IPA), grouped according to their primary biological function categories during the progression and intervention phases based on the global analysis.

Differentially expressed genes (DEGs) were identified from two pairwise comparisons: P-HC24 versus P-LC24 (Progression, Prog), and I-HC32 versus I-LC32 (Intervention, Int). DEGs were determined both at the global level, encompassing all cells, and at the level of major hepatic cell populations (Figure 2A). Only genes that were significant concerning differential expression (adjusted *p*-value < 0.1) were included in further analysis. At the global level, high cholesterol intake upregulated 2,572 genes and downregulated 2,195 genes, whereas cholesterol reduction upregulated 3,702 genes and downregulated 724 genes (Figure 2A). We identified 278 genes that reversed their direction of expression in response to dietary cholesterol, with 75 genes upregulated during the progression phase and subsequently downregulated following dietary intervention, and 203 genes downregulated by high cholesterol intake and upregulated after cholesterol reduction (Figure 2B). The 75 genes upregulated by high cholesterol intake enriched Gene Ontology (GO) pathways related to oxidative phosphorylation and energy production (Figure 2C, top). Conversely, the 203 genes upregulated after dietary intervention enriched GO terms associated with macromolecule regulation, gene expression, and platelet-activating factor (Figure 2C, bottom). Additionally, high cholesterol intake for 24 weeks modulated 181 pathways, while cholesterol reduction for 8 weeks affected 265 pathways (Figure 2D). The majority of pathways modified by dietary intervention were related to immune response and inflammation. Additional categories included cell survival and apoptosis, cell cycling regulation, protein/RNA/DNA synthesis, mitochondrial stress, lipid and carbohydrate metabolism, and detoxification.

### 3.2 Longitudinal profiling reveals intervention-driven transcriptomic shifts in hepatic innate and adaptive immune cells

The transcriptomes of innate immune cells were moderately affected by cholesterol intake in the context of the FPC diet. High cholesterol intake altered the expression of 97 genes in KCs, 261 genes in monocytes, 22 genes in neutrophils, and 91 genes in NK cells (Figure 2A). Reducing dietary cholesterol affected the expression of 134 genes in KCs, 172 genes in monocytes, and only 10 genes in NK cells. Prolonged intake of high cholesterol induced robust upregulation of several immune-related pathways in Kupffer cells and monocytes and, to a lesser extent, in NK cells, with minimal impact on neutrophil pathways. Notably, cholesterol reduction reversed most of the pathways upregulated during the progression phase.

**Figure 3:**
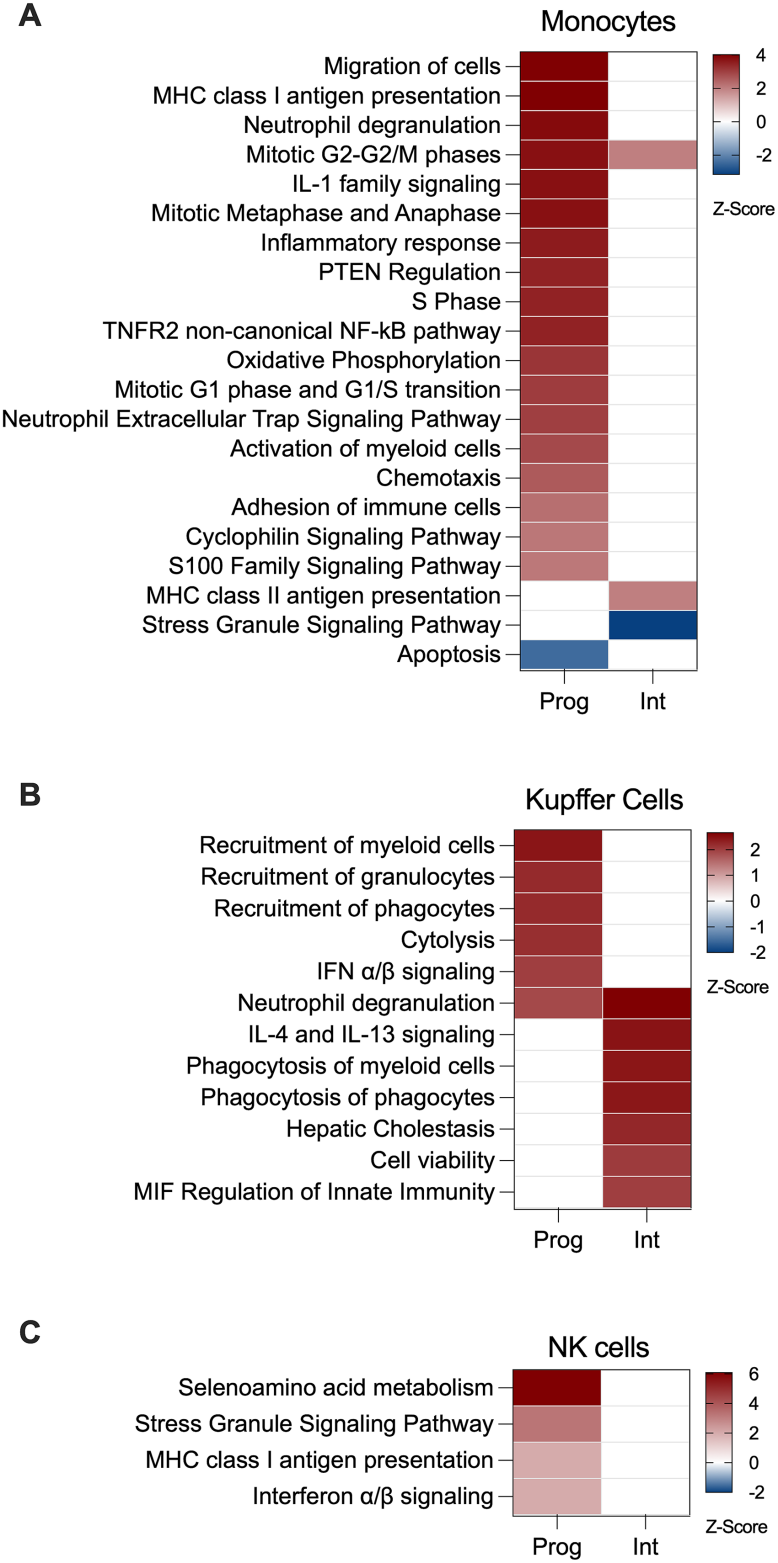
Transcriptional reprogramming of hepatic innate immune cells following dietary intervention. **(A)** The heatmap shows robust upregulation of pathways related to inflammation and cell proliferation in monocytes from mice fed the FPC high-cholesterol diet. These pathways normalized following dietary intervention. **(B)** During the progression phase, pathways associated with cell recruitment and inflammation were upregulated in Kupffer cells, whereas dietary cholesterol reduction upregulated pathways involved in phagocytosis and cell viability. **(C)** Natural killer cells were less affected by the high-cholesterol diet in the context of the FPC diet. Pathways and biological processes identification were performed using Ingenuity Pathway Analysis (IPA). Prog: Progression phase; Int: Intervention phase. NK cells: Natural killer cells.

Monocytes displayed the largest number of pathways activated by the high-cholesterol FPC diet (Figure 3A), primarily involving cell chemotaxis and recruitment, cell activation and proliferation, neutrophil degranulation, and inflammation. Most of these pathways were deactivated following dietary intervention, while MHC-II-mediated antigen presentation became activated. In Kupffer cells, the most significantly upregulated pathways during progression phase were linked to granulocyte and myeloid cell recruitment, cytolysis, neutrophil degranulation, and IFN-*α*/*β* signaling (Figure 3B). Neutrophil degranulation signaling persisted after dietary intervention, and phagocytosis, IL-4/IL-13 signaling, as well as cell viability pathways were further upregulated after dietary cholesterol reduction. Natural killer cells showed activation of only a small number of pathways during the progression phase, including stress granule signaling, MHC-I-mediated antigen presentation, and IFN-α/β signaling (Figure 3C). All these pathways were normalized after cholesterol reduction.

In contrast to innate cells, adaptive immune cells were substantially more sensitive to the levels of cholesterol in the FPC diet, displaying the highest numbers of DEGs (Figure 2A). High cholesterol intake altered the expression of 617 genes in CD8^+^ T cells, 865 genes in CD4^+^ T cells, 1,872 genes in naïve B cells, 1,452 genes in memory B cells, and 136 genes in plasmablasts. Dietary intervention modulated the expression of 440 genes in CD8^+^ T cells, 292 genes in CD4^+^ T cells, 839 genes in naïve B cells, 1,032 genes in memory B cells, and 627 genes in plasmablasts.

**Figure 4:**
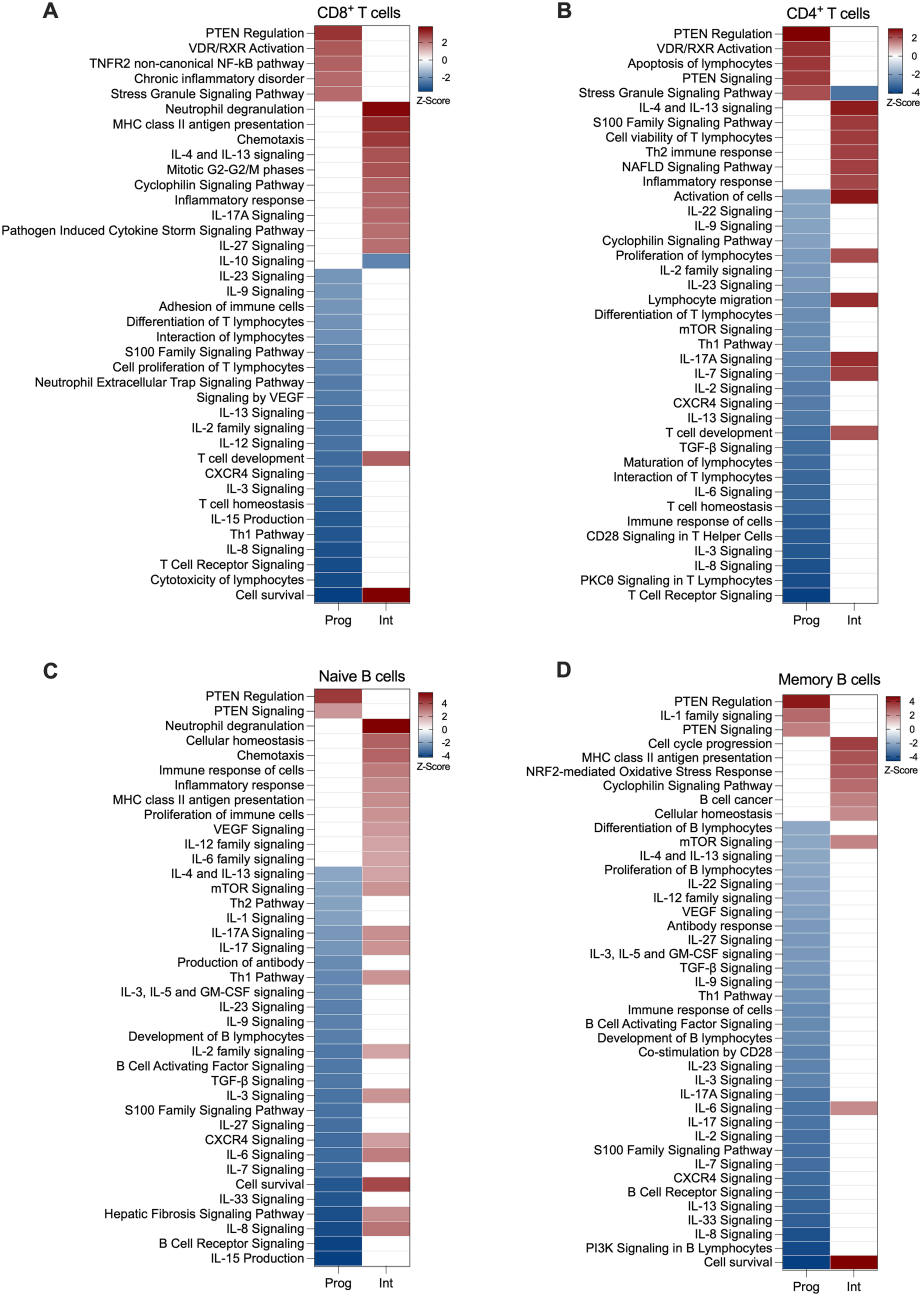
Dynamic remodeling of hepatic adaptive immune cell transcriptomes during disease progression and dietary intervention. The FPC high-cholesterol diet negatively impacted immune-related pathways in CD8^+^ T cells **(A)**, CD4^+^ T cells **(B)**, naïve B cells **(C)**, and memory B cells **(D)**. Cholesterol removal normalized adaptive immune cell transcriptomes and upregulated several inflammatory pathways. Pathways and biological processes identification were performed using Ingenuity Pathway Analysis (IPA). Prog: Progression phase; Int: Intervention phase.

Most pathways in T cells were downregulated during the progression phase. Following cholesterol reduction, the majority normalized or became upregulated. In CD8^+^ T cells, high cholesterol activated PTEN regulation, VDR/RXR signaling, and TNFR2 non-canonical NF-kB pathway while downregulating homeostasis-related pathways, such as IL-2 signaling, T cell receptor (TCR) signaling, cell survival, development, and differentiation, and several pro-inflammatory pathways including IL-8 signaling and Th1 response (Figure 4A). Dietary intervention upregulated all homeostasis-related pathways as well as several pro-inflammatory cytokine signaling cascades. High cholesterol also upregulated apoptosis, PTEN signaling and regulation, and VDR/RXR activation in CD4^+^ T cells, while downregulating IL-2 signaling, TCR signaling, and CD28 and PKCθ signaling (Figure 4B). Cholesterol reduction normalized or upregulated homeostasis-related pathways and activated pathways involved in cell viability, the NAFLD signaling pathway, and the Th2 response.

Naïve and memory B cells showed similar pathway modulation patterns (Figures 4C,D). During the progression phase, both B cell populations demonstrated upregulation of PTEN regulation, and strong downregulation of IL-8, IL-7, IL-9, IL-27 signaling, CXCR4 signaling, B cell receptor signaling, B cell activating factor signaling, B cell development, and the Th1 pathway, among others. In the intervention phase, most of these pathways normalized or were upregulated. Plasmablasts exhibited only weak pathway enrichment (data not shown).

## 4 Discussion

Traditional assays used in animal studies, such as bulk RNA-seq and fluorescence-activated cell sorting (FACS), typically require terminal sampling procedures to obtain large amounts of tissue and often demand a substantial number of animals per experimental condition. Here, we demonstrate that image-guided fine-needle aspiration (FNA), coupled with single-cell RNA sequencing (scRNA-seq), offers a safe and powerful non-terminal sampling method that overcomes these limitations in MASLD and MASH animal studies. This approach is minimally invasive and enabled the longitudinal characterization of disease trajectories at single-cell resolution using significantly fewer animals, while allowing both identification and transcriptomic profiling of hepatic immune cell populations. All mice undergoing repeated sampling fully recovered without complications, and the method consistently yielded a robust number of viable cells suitable for single-cell transcriptomics.

We investigated the utility of this approach to assess the impact of dietary cholesterol on hepatic immune cells during MASH progression in a model of diet-induced MASH. Although histological assessment did not reveal significant differences in disease severity among the groups (Supplementary Table 1), we were able to comprehensively characterize the hepatic innate and adaptive immune landscapes during MASH progression and after dietary intervention. Our findings are consistent with the existing MASLD literature, particularly regarding monocytes and Kupffer cells (KCs), which are well-established contributors to MASLD pathogenesis (13, 22, 23). KCs, the liver-resident macrophages, can display two major phenotypes: pro-inflammatory (or M1) and anti-inflammatory/tissue remodeling (or M2). Steatosis and consequent liver injury modify the M1/M2 balance, advancing hepatic chronic inflammation (steatohepatitis) (24). Monocytes are commonly recruited to the liver during inflammation when the KC pool is depleted, maturing into monocyte-derived macrophages that secrete large amounts of pro-inflammatory mediators, and contribute to steatohepatitis and fibrosis progression (24). Previously, we have shown that prolonged high cholesterol intake combined with a high-fat diet increases hepatic inflammation, elevates the numbers of pro-inflammatory infiltrating macrophages, and leads to a dysfunctional M2 Kupffer cell population with pro-fibrotic traits (13, 22). Our results using the FNA-scRNA-seq approach support that high cholesterol intake induces strong activation of the inflammatory pathway in Kupffer cells and monocytes during MASH progression, many of which normalize following dietary intervention.

Conversely, the role of adaptive immune cells in MASLD is relatively underexplored, but growing evidence suggests that they are essential for MASLD development. T cell subsets have a complex role in MASH pathogenesis, in which they may exacerbate liver inflammation, leading to hepatocyte damage and fibrosis (25). Intrahepatic B cells can be found in higher numbers in MASH patients and in mice fed a high-fat diet, and may contribute to MASLD-to-MASH progression through multiple mechanisms, including interaction with T cells, activation of hepatic stellate cells (HSC) and KCs, and secretion of antibodies, and pro-inflammatory and pro-fibrotic factors (26). We observed that T and B cells exhibited greater transcriptomic plasticity, with high cholesterol intake inhibiting multiple homeostatic pathways during disease progression. Dietary cholesterol reduction led to substantial reactivation of the same pathways.

Our approach enabled simultaneous transcriptomic profiling of adaptive and innate immune cells, and our findings highlight the distinct temporal sensitivities of innate and adaptive immune compartments, which can often be masked in traditional cross-sectional designs. A major limitation of this framework is that not all immune cell types were identified in our datasets. Although we detected a few neutrophils, we could not detect a considerable number of other granulocytes. These cells have a short lifespan ex vivo and are particularly sensitive to tissue digestion, which can lead them to activation or apoptosis (27). On the other hand, we detected an unusually high proportion of B cells compared to myeloid cells (28, 29). Although intrahepatic B-cell accumulation has been reported in experimental models of MASH and may be associated with disease progression (26), their abundance may also have been influenced by other factors, such as the non-parenchymal cell (NPC) isolation method. Additionally, we were unable to detect other NPC populations potentially involved in MASLD-to-MASH progression, such as liver sinusoidal endothelial cells (LSECs) (30) and HSCs (31). Together, these biases in cell recovery may have affected downstream gene expression analyses. The unbalanced representation of hepatic cell populations also limits the interpretation of the global gene expression comparisons, as changes in transcript abundance may be driven, at least in part, by the relative cell-type composition of the samples. This highlights the importance of incorporating scRNA-seq approaches to resolve cell-type-specific transcriptomic changes and provide a more comprehensive characterization of the samples. While we cannot determine the exact source of the observed cell enrichment or depletion, we believe that the liver tissue dissociation method used for NPC isolation may have contributed to the observed cell composition rather than the FNA procedure itself. Previous studies have shown that liver tissue dissociation methods can affect the composition of recovered cell populations, particularly when comparing *ex vivo* digestion and *in vivo* liver perfusion (32), indicating that cell ratios obtained from single-cell analyses do not necessarily reflect the true cellular composition of the liver. This limitation may be addressed by optimizing tissue digestion protocols and/or applying complementary transcriptomic approaches, such as single-nucleus RNA sequencing. Another limitation was the need to pool samples to obtain sufficient numbers of high-quality cells for scRNA-seq. Dissociation of samples from individual animals resulted in poor cell yield. Future implementations incorporating sample barcoding prior to pooling would enable *post hoc* demultiplexing and inclusion of biological replicates (12). Despite these limitations, our results position the FNA-scRNA-seq framework as a versatile and scalable platform for longitudinal immune profiling in preclinical MASH studies. This approach could be readily expanded to evaluate therapeutic responses, capture early disease events, and enable temporal mapping of pathway rewiring across diverse liver-resident immune-stromal interactions.

## Data Availability

The datasets presented in this study can be found in online repositories. The names of the repository/repositories and accession number(s) can be found at: https://www.ncbi.nlm.nih.gov/geo/query/acc.cgi?acc=GSE320221.

## In memoriam

This manuscript is dedicated to the memory of Hugo R. Rosen, who inspired a love for immunology and hepatology and fostered a spirit of teamwork and respect for all. Rosen initiated this study and contributed to it significantly.
