# Supplementary material for: Integrating fine needle aspiration and single-cell RNA sequencing for studying metabolic dysfunction-associated steatotic liver disease
Source: Front Med (Lausanne). 2026 Jul 20;13:1817738. doi: 10.3389/fmed.2026.1817738 (PMC13429742; doi:10.3389/fmed.2026.1817738)
Supplement: Supplementary file 1 [file Data_Sheet_1.DOCX]

Integrating fine needle aspiration and single-cell RNA sequencing for studying metabolic dysfunction-associated steatotic liver disease

Matthew P. Salomon^1&^, Lucy Golden-Mason^2&^, Ivetta Vorobyova^3^, Gary C. Kanel^4^, Yufen Wang^2^, Daphne Wong^1^, Ana C. Maretti-Mira^2*^

^1^Department of Cancer Biology, Keck School of Medicine, University of Southern California, Los Angeles, CA, United States

^2^Division of Gastrointestinal and Liver Diseases, Department of Medicine, Keck School of Medicine, University of Southern California, Los Angeles, CA, United States

^3^Molecular Imaging Center, Department of Radiology, Keck School of Medicine, University of Southern California, Los Angeles, CA, United States

^4^Department of Pathology, Keck School of Medicine, University of Southern California, Los Angeles, CA, United States

^&^Joint First Authors

**Content List:**

Supplementary Table 1

Supplementary Table 2

Supplementary Figure 1

Supplementary Figure 2

Supplementary Figure 3

Supplementary Figure 4

| **Supplementary Table 1: Histological findings** | | | | |
| --- | --- | --- | --- | --- |
| **Sub-group** | **Replicate** | **Macrovesicular Steatosis** | **Lobular**  **Inflammation** | **Fibrosis** |
| P-LC24 | 1 | 4 | 1 | 2 |
|  | 2 | 4 | 1 | 2 |
| P-HC24 | 1 | 4 | 2 | 2 |
|  | 2 | 4 | 1 | 1 |
| I-LC32 | 1 | 4 | 2 | 2 |
|  | 2 | 4 | 2 | 2 |
|  | 3 | 4 | 1 | 2 |
| I-HC32 | 1 | 4 | 1 | 2 |
|  | 2 | 4 | 1 | 2 |
|  | 3 | 4 | 2 | 2 |
| Parameters used for analysis: Steatosis:  0: None  <1+: less than 5% of hepatocytes  1+: 5-25%  2+: 26-50%  3+: 51-75%  4+: >75%  Lobular inflammation (clusters, 20x):  0: None  1+: <2 foci  2+: 2-4 foci  3+: >4 foci  Fibrosis:                0: None                1: Perisinusoidal or periportal                1a: Mild, zone 3, perisinusoidal                1b: Moderate, zone 3, perisinusoidal                1c: Portal/periportal                2: Perisinusoidal and portal/periportal                3: Bridging fibrosis                4: Cirrhosis | | | | |

| **Supplementary Table 2: Number of viable cells recovered from FNA biopsies** | | | | |
| --- | --- | --- | --- | --- |
| **Phase** | **Sub-group** | **Diet** | **# cells** | **Viability** |
| Progression | P-LC24 | FPC + 0.05% Chol – 24wk | 49,200 | 100% |
|  | P-HC24 | FPC + 1.2% Chol – 24wk | 559,000 | 100% |
| Intervention | I-LC32 | FPC + 0.05% Chol – 24wk  FPC + 0.05% Chol – 8wk | 137,000 | 90% |
|  | I-HC32 | FPC + 1.2% Chol – 24wk  FPC + 0.05% Chol – 8wk | 206,850 | 93% |


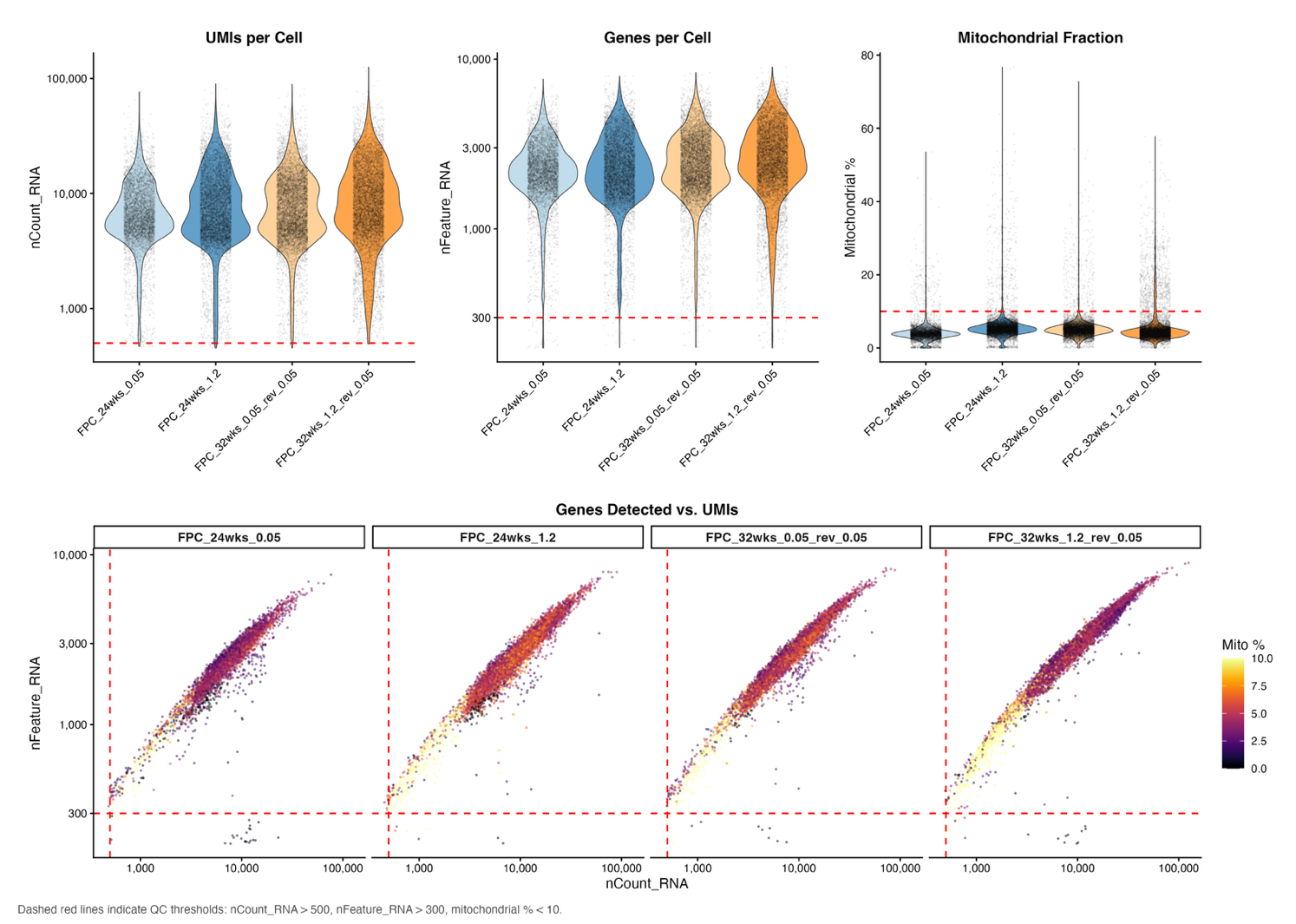


**Supplementary Figure 1. Quality control thresholds applied during raw bioinformatic data processing.** Low-quality cells were defined as those with fewer than 500 UMIs, fewer than 300 detected genes, or more than 10% mitochondrial reads, and were excluded from further analysis. Genes detected in fewer than three cells were also removed from the datasets. Red dotted lines indicate the applied thresholds.


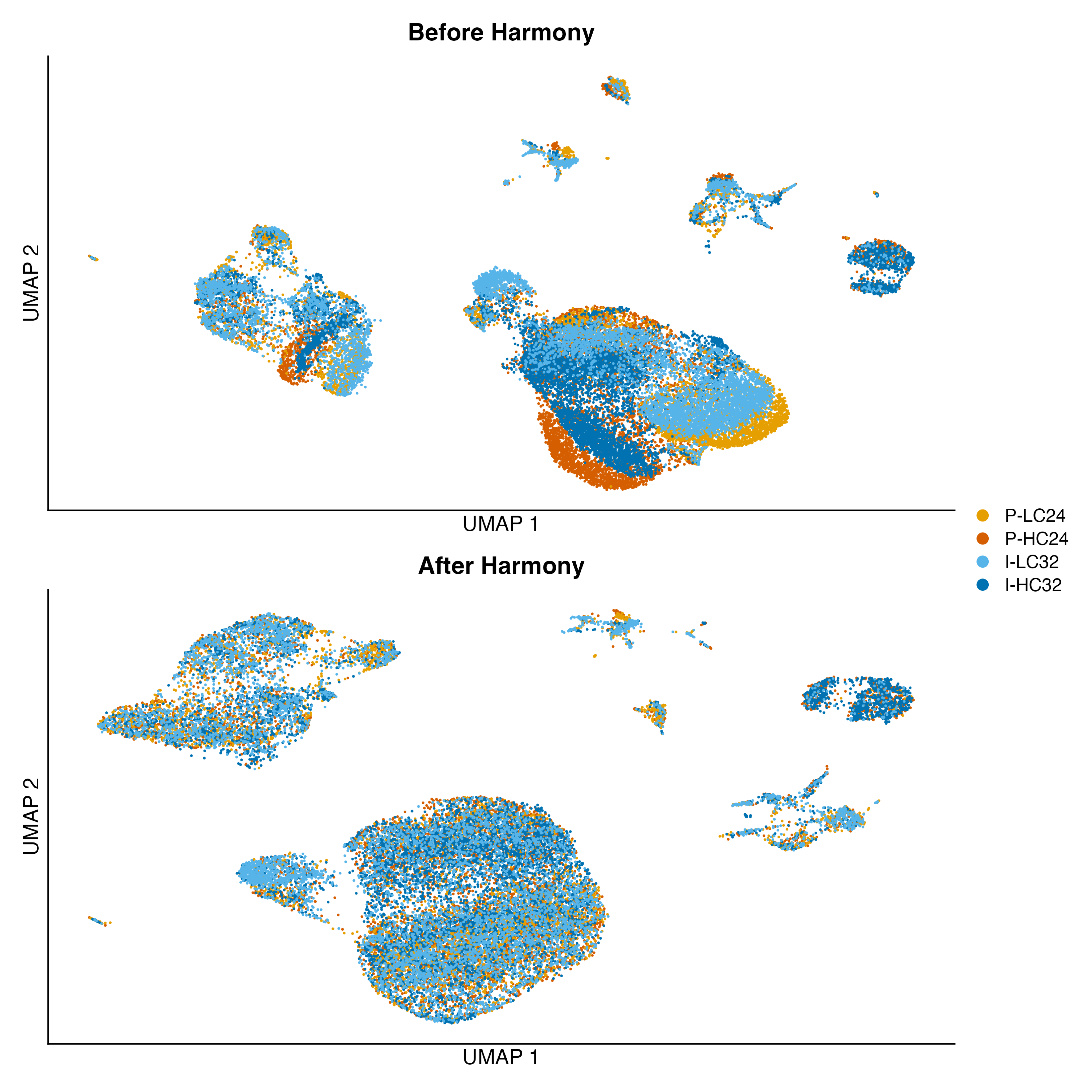


**Supplementary Figure 2. Harmony dataset integration.** Harmony integration was performed on the principal component embedding with each scRNA-seq library treated as a separate batch to correct the library-level technical variation across the four samples. No biological covariates were included in the correction. Integration performance was assessed by comparing UMAP embeddings before and after Harmony, colored by condition.
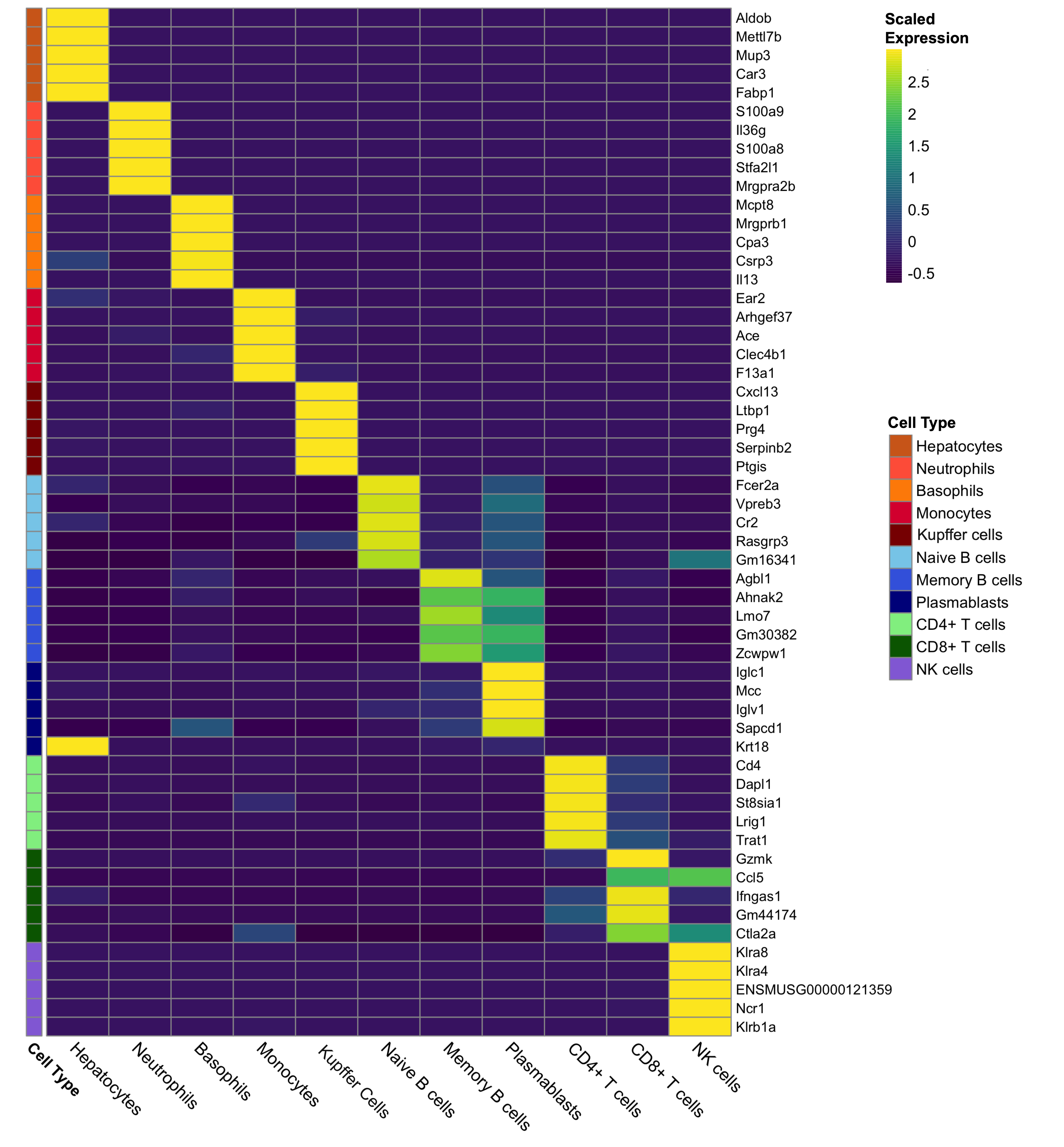


**Supplementary Figure 3. Canonical marker genes used for cell annotation.** Cell types were manually annotated by examining the expression of canonical marker genes across each cluster.


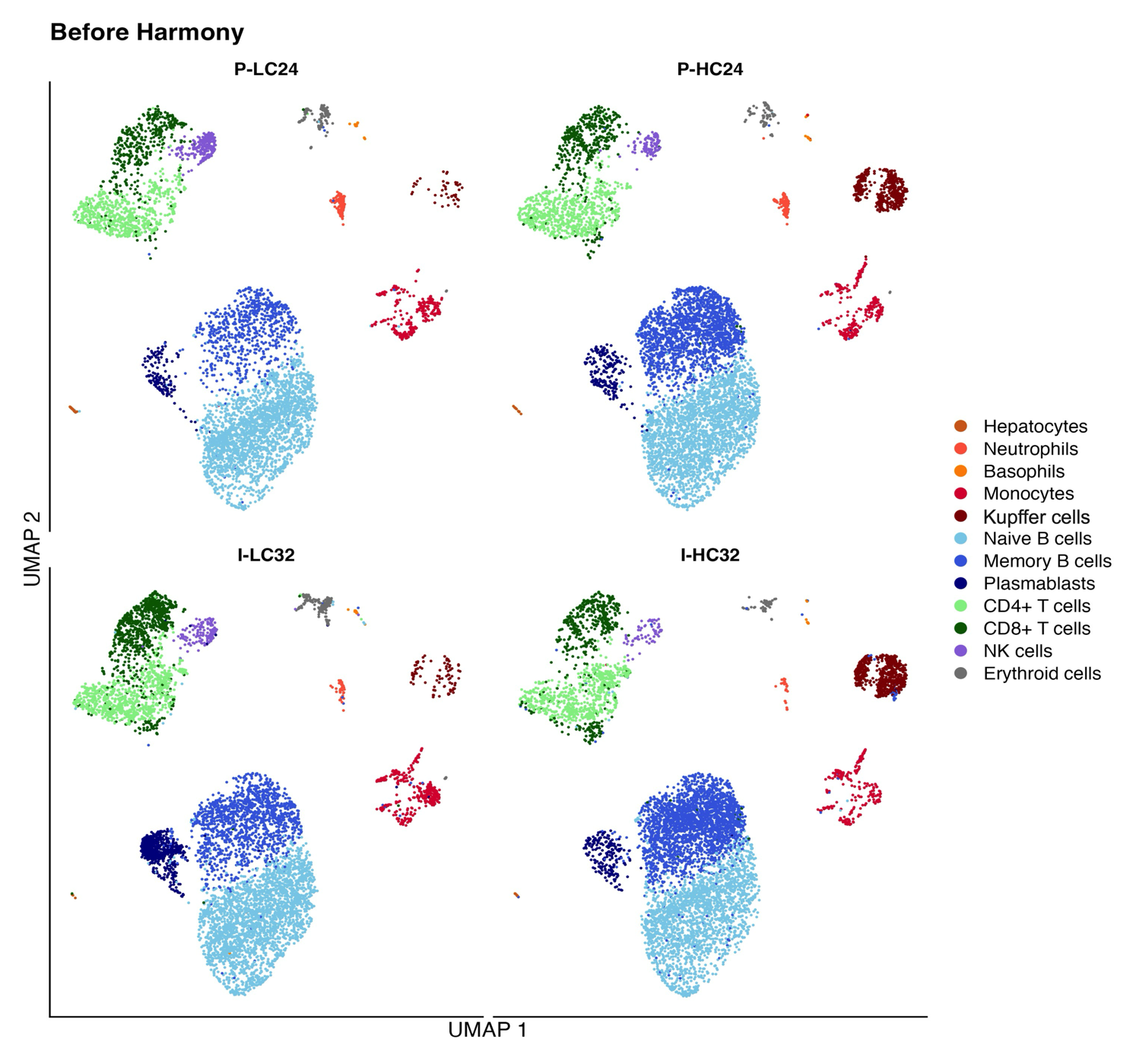


**Supplementary Figure 4. UMAP projection of cell clusters.** Cell clusters from each sample included in this study projected into two-dimensional space using Uniform Manifold Approximation and Projection (UMAP) before dataset integration by Harmony.
